# Supplementary material for: Periodic corner holes on the Si(111)-7×7 surface can trap silver atoms
Source: Nat Commun. 2022 May 27;13:2973. doi: 10.1038/s41467-022-29768-6 (PMC9142567; doi:10.1038/s41467-022-29768-6)
Supplement: Supplementary file 1 — Supplementary Information [file 41467_2022_29768_MOESM1_ESM.pdf]

Supplementary Information: Periodic corner holes on the Si(111)-7x7 surface can trap silver atoms

Jacek R. Osiecki[1,2]*, Shozo Suto[2], Arunabhiram Chutia[3]*

Affiliations

[1] MAX IV Laboratory, Lund University SE22100, Lund, Sweden

[2]Department of Physics, Tohoku University, Sendai, 980-8578, Japan.

[3]School of Chemistry, University of Lincoln, Brayford Pool, LN6 7TS, UK.

*Corresponding author. Email: **jacek.osiecki@maxiv.lu.se** and **achutia@lincoln.ac.uk**

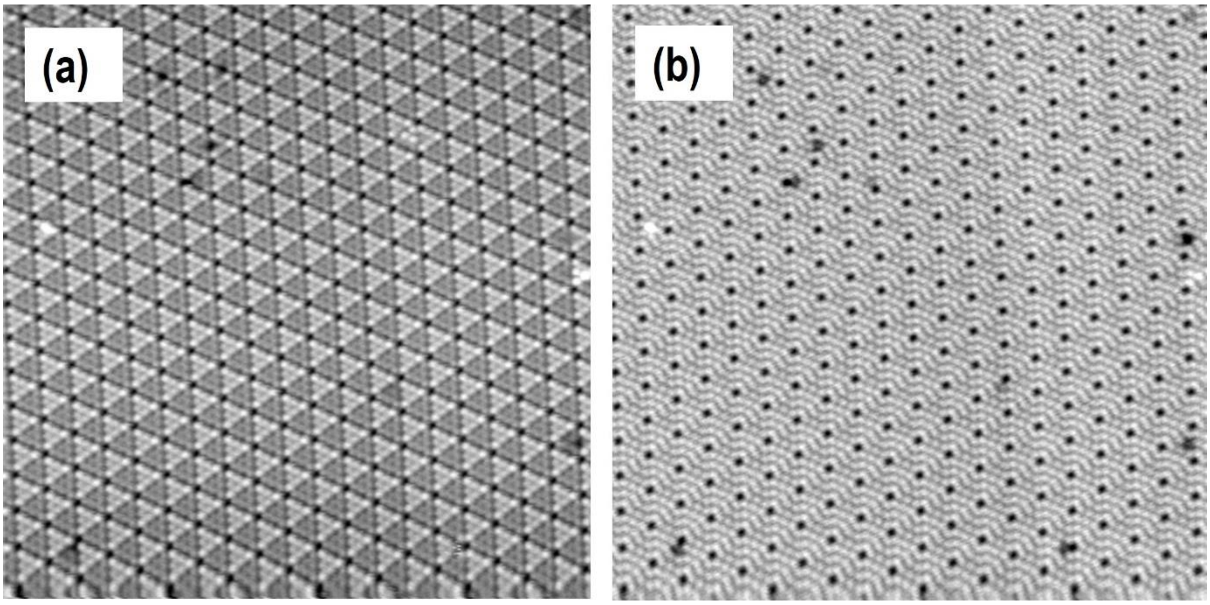

Supplementary Figure 1: Clean Si(111)7x7. The STM images of the Si(111)-7x7 surface of the same area, filled states in (a) and empty states in (b). Tunneling conditions i.e. current is 150 pA and voltage is ±1.93V. The 7x7 surface has low defect density. Size of the scanned area is 50 nm x 50 nm. Images in (a) and in (b) are in the same file**:** m14_ori.par , m14_ori.tf0, m14_ori.tb0 in Supplementary Data 2. Files are in original STM Omicron format.

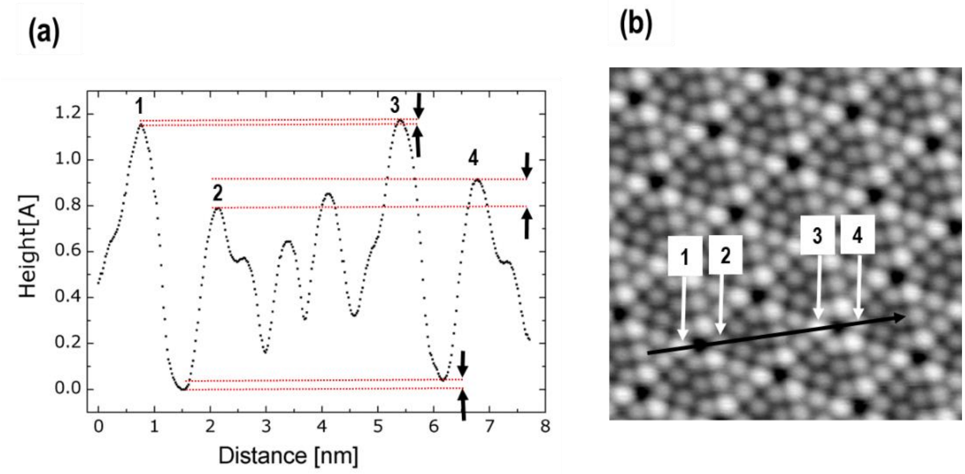

Supplementary Figure 2: Cross section through two CHs. (a) A cross section through two corner holes with and without Ag atom inside along the line as shown in image in (b) (the black line with the arrow). (b) The filled states STM image (-2.0 V, 153 nA) with one corner hole occupied with the Ag atom inside between adatoms 3 and 4. Six adatoms that are around the corner hole with Ag atom looks brighter than the empty ones. The bottom of the corner hole with Ag is 5 picometers higher than the empty one. If the adatoms surrounds CH with Ag atom inside they are higher by about 2 to 10 pm depending on the adatom correspondingly. Image in (b): m63_ori.par, m63_ori.tf0, m63_ori.tb0 in Supplementary Data 2 Files are in original STM Omicron format.

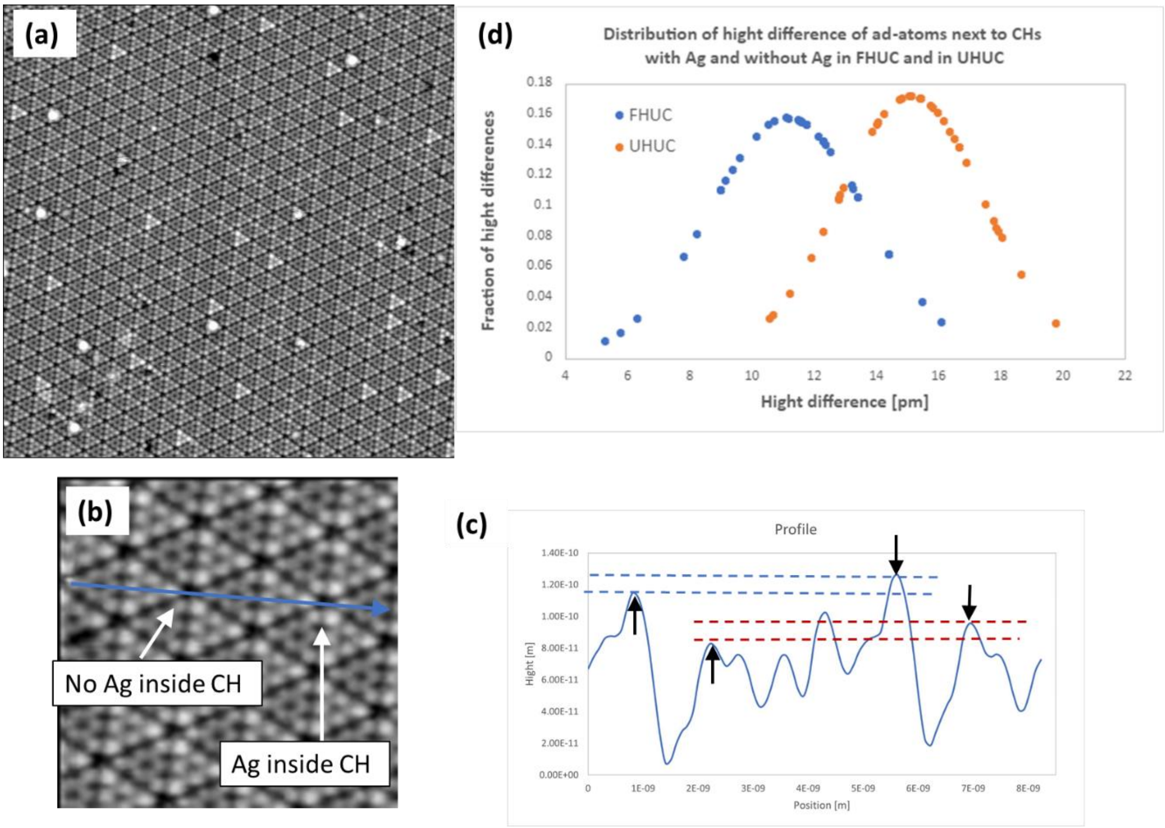

Supplementary Figure 3: Height change of adatoms around CHs with Ag atom. a) Filled states STM image of the Si(111)-7x7 surface with 58.1 nm x 58.1nm with Ag atoms, obtained at -2.2 V and 800 pA. b) zoom in image 10nm x 11.3 nm obtained from a). The blue arrow in b) denotes the position of cross section in c). c) typical cross section through corner adatoms and two adjacent CHs with Ag and without Ag inside. The cross section shows the high difference between adatoms next to corner holes with Ag and without Ag in FHUC and UHUC. d) Graph showing the distribution of the height differences of 35 random adatoms next to corner holes with Ag and without in FHUCs (blue line) and UHUCs (orange line). The average height difference between adatoms in FHUC is 11.15 ± 2.54 pm and in UHUCs is 15.13 ± 2.33 pm. The height distribution is a normal gauss distribution indicating existence of one height value for Ag with a standard deviation related to measurement error. In order to fill more CHs with the Ag atoms and obtain higher surface density of Ag in CHs, the deposition was performed at 91 Celsius degrees. Temperature was measured in a proximity of the sample with the thermocouple. The deposition lasted 8 minutes. The temperature of the sample during scanning with STM was 330 K. Image in (a): default_2021Dec06-130754_STM-STM_Spectroscopy--2_4.Z_mtrx, default_2021Dec06-130754_STM-STM_Spectroscopy_0001.mtrx in Supplementary Data 2 Files are in original STM Omicron format.

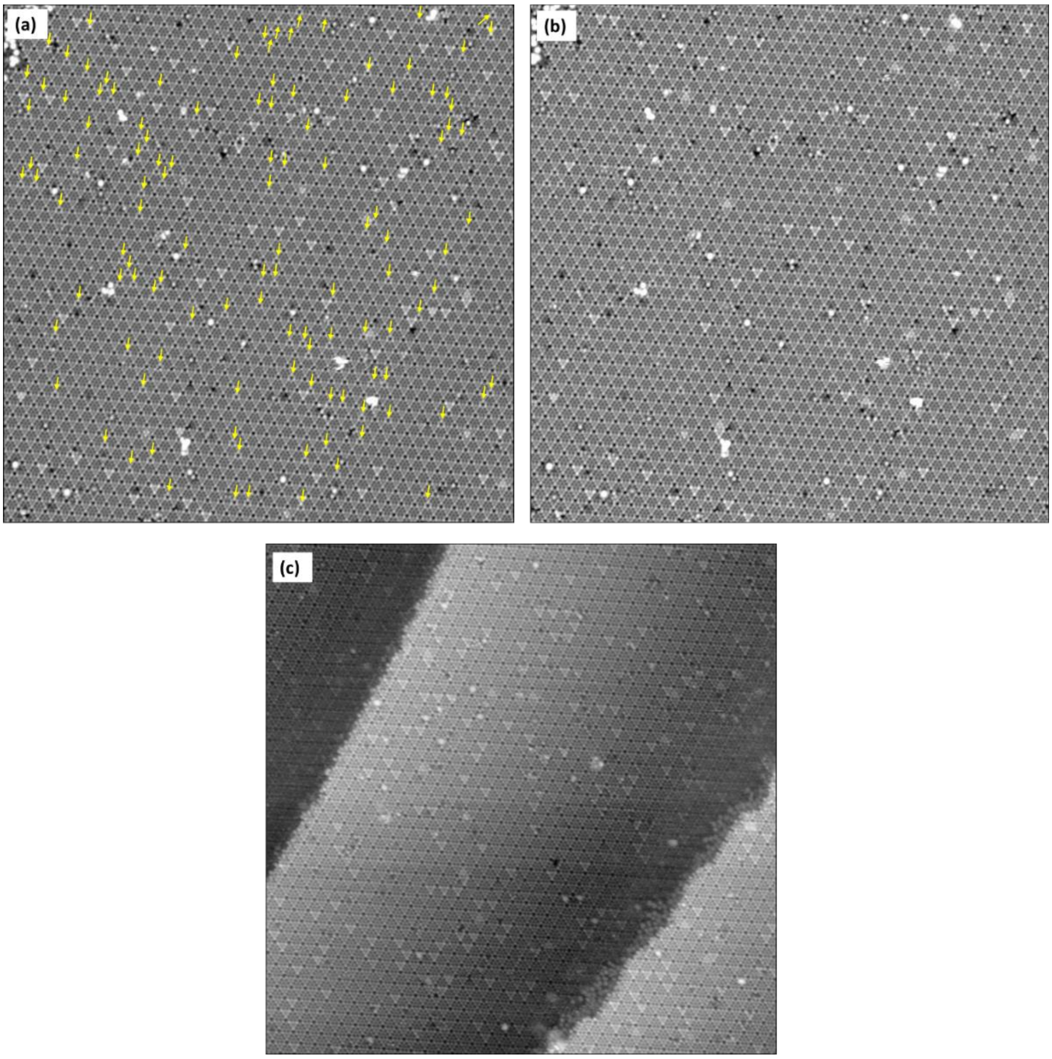

Supplementary Figure 4: No tip influence on the Ag atom in CH. Filled states STM images a) and b) of the same area of the Si(111)-7x7 surface 100 nm x 100nm, obtained at -1.8 V and 200 nA. The tip in between frames a) and b) was retracted 1µm for 1h and after that the contact was reestablished. In the image a) there are 116 Ag atoms inside CHs denoted by the arrows. In the image b) we can see that all of the Ag atoms inside CH denoted in a) remained in the CHs after 1h with retracted STM tip. In order to fill more CHs with the Ag atoms, and obtain higher surface density and better statistics, the deposition was performed at 100 ± 20 Celsius degrees. We have obtained 116 Ag inside CH as in a) and b). If the same amount of Ag is deposited at RT much less of corner holes will be filled with Ag as seen in c) (size 100 nm x 100nm, -1.8V, 200nA). There are more Ag atoms outside CHs in c) and majority of Ag atoms reside in FHUCs. Image in (a): default_2021Nov09-143943_STM-STM_Spectroscopy_0001.mtrx, default_2021Nov09-143943_STM-STM_Spectroscopy--23_2.Z_mtrx. Image in (b): default_2021Nov09-143943_STM-STM_Spectroscopy_0001.mtrx, default_2021Nov09-143943_STM-STM_Spectroscopy--29_1.Z_mtrx in Supplementary Data 2, Files are in original STM Omicron format.

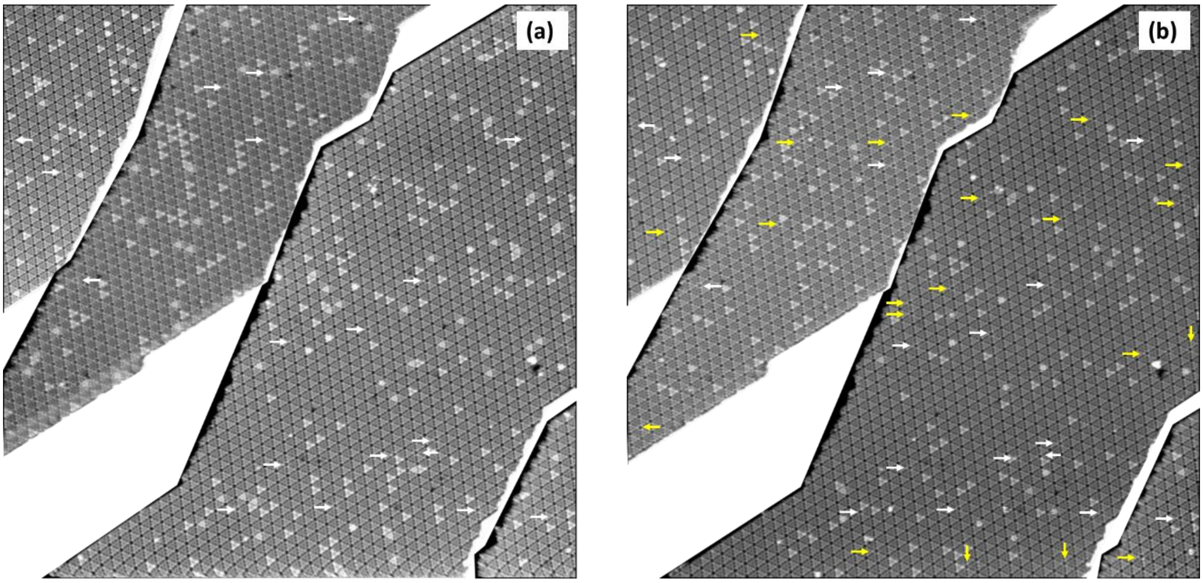

Supplementary Figure 5: Stability of Ag inside CH at RT. Two STM images (a) and (b) of the same area (100 nm x 100 nm, -2.0 V, 150 nA). The images were modified and height level of each terrace is brought to the same level. Image in (a) was acquired at the beginning of the experiment and image in (b) was acquired after 4 days and 7 hours of continuous scanning. White arrows point to the CH with Ag atoms that are inside CHs from the beginning of the experiment. Yellow arrows point to the CH that become occupied with Ag during the whole scanning time. Image in (a): m23_ori.par, m23_ori.tf0. Image in (b): m1228_ori.par, m1228_ori.tf0 in Supplementary Data 2, Files are in original STM Omicron format.

Supplementary Figure 6.

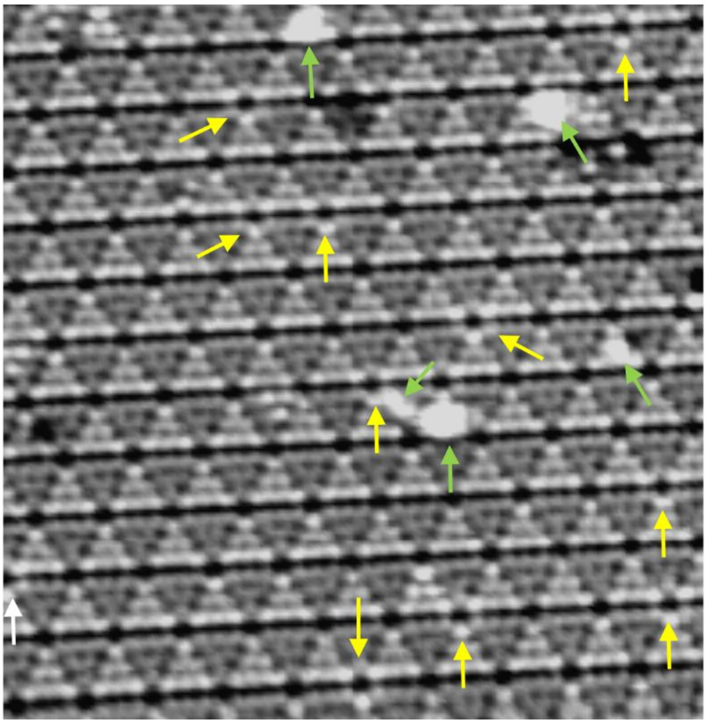

Supplementary Figure 6. Si(111)-7x7 at 150 °C with Ag atoms. Filled states STM image of the Si(111)-7x7 surface (-1.0V, 166nA). There are some clusters in HUCs that trapp atoms at 150 °C denoted with green arrows. Corner holes with Ag atoms inside denoted with yellow arrows. The corner holes and clusters in HUCs are main long time "trapping units" of single Ag atoms especially at 150 °C.

Supplementary Table 1

The lifetime in HUC and hopping rate to the hopping to corner hole vs temperature.

Jacek Osiecki, Atomistic diffusion and clustering of Ag atoms on a well-defined Si(111)7×7 surface studied by STM, *PhD Thesis*, (2007).

Temperature [°C]	Lifetime in HUC [s]	Hopping rate to corner hole [1/s]
54.2	4.31E+04	2.32E-05
58.9	3.67E+04	2.73E-05
52	3.34E+04	3.00E-05
45	6.91E+04	1.45E-05
37.9	2.36E+05	4.23E-06
34	2.40E+05	4.16E-06
25	4.82E+05	2.07E-06
18.1	1.58E+06	6.33E-07
11.8	2.94E+06	3.40E-07

Supplementary Table 2

Frequency pre-factor and the activation energy for the jump of the Ag atom to the CH.

Jacek Osiecki, Atomistic diffusion and clustering of Ag atoms on a well-defined Si(111)7×7 surface studied by STM, *PhD Thesis*, (2007).

	$\nu^0_{F,U}$ frequency prefactor [1/s]	$E_{F,U}$ activation energy [eV]
**Jump to corner hole**	**1.4E+8**	**0.828**
