# Supplementary material for: Periodic corner holes on the Si(111)-7×7 surface can trap silver atoms
Source: Nat Commun. 2022 May 27;13:2973. doi: 10.1038/s41467-022-29768-6 (PMC9142567; doi:10.1038/s41467-022-29768-6)
Supplement: Supplementary file 3 — Description of Additional Supplementary Files [file 41467_2022_29768_MOESM3_ESM.docx]

**Supplementary data files description: Periodic corner holes on the Si(111)-7x7 surface can trap silver atoms**

Jacek R. Osiecki^1,2^*, Shozo Suto^2^[, Arunabhiram Chutia](https://pubs.rsc.org/en/results?searchtext=Author%3AArunabhiram%20Chutia)^3^*

**Affiliations**

^1^ MAX IV Laboratory, Lund University SE22100, Lund, Sweden

^2^Department of Physics, Tohoku University, Sendai, 980-8578, Japan.

^3^School of Chemistry, University of Lincoln, Brayford Pool, LN6 7TS, UK.

*Corresponding author. Email: **jacek.osiecki@maxiv.lu.se** and **achutia@lincoln.ac.uk**

File name: **Supplementary Data 1.zip**

Description: Zip folder contains all files of coordinates of atomic positions obtained in the calculations. The files are provided for Cu, Au, Li, Na, F, I and Ag. Atomic positions are also provided for cluster model. The files are placed in separate folders with proper names e.g: folder a_Cu_OK contains files for Cu atom.

File name: **Supplementary Data 2.zip**

Description: Zip folder contains original files used to make supplementary figures and figure 5 in the main manuscript.

The list of the files in Supplementary Data 2:

- m14_ori.par , m14_ori.tf0, m14_ori.tb0.
- m63_ori.par , m63_ori.tf0, m63_ori.tb0.
- default_2021Dec06-130754_STM-STM_Spectroscopy--2_4.Z_mtrx, default_2021Dec06-130754_STM-STM_Spectroscopy_0001.mtrx
- m23_ori.par , m23_ori.tf0, m1228_ori.par , m1228_ori.tf0
- default_2021Nov09-143943_STM-STM_Spectroscopy_0001.mtrx, default_2021Nov09-143943_STM-STM_Spectroscopy--23_2.Z_mtrx, default_2021Nov09-143943_STM-STM_Spectroscopy_0001.mtrx, default_2021Nov09-143943_STM-STM_Spectroscopy--29_1.Z_mtrx
- DOS_7x7_5x5_AgSi111.xlsx
